# Lactate, a putative survival factor for myeloma cells, is incorporated by myeloma cells through monocarboxylate transporters 1

**DOI:** 10.1186/s40164-015-0008-z

**Published:** 2015-04-21

**Authors:** Shiho Fujiwara, Naoko Wada, Yawara Kawano, Yutaka Okuno, Yoshitaka Kikukawa, Shinya Endo, Nao Nishimura, Nina Ueno, Hiroaki Mitsuya, Hiroyuki Hata

**Affiliations:** Department of Hematology, Kumamoto University School of Medicine, 1-1-1, Honjo, Chu-ouku, Kumamoto city, Kumamoto 860-8556 Japan; Graduate School of Health Sciences, Faculty of Medical Sciences, Division of Informative Clinical Sciences, Kumamoto University School of Medicine, 4-24-1 Kuhonji, Kumamoto, 862-0976 Japan

**Keywords:** Multiple myeloma, Glucose, Lactate, Monocarboxylate transporters

## Abstract

**Background:**

Lactate levels within tumors are correlated with metastases, tumor recurrence, and radioresistance, thus apparently contributing to poor outcomes in patients with various cancers. We previously reported that high-level production of lactate by multiple myeloma (MM) cell lines is associated with high-level LDH activity within such MM cells. However, the kinetics of lactate remains to be studied. In the present study, we attempted to elucidate the mechanism of lactate incorporation into MM cells.

**Methods:**

Six MM cell lines and stromal cells obtained through long-term culture of bone marrow samples from MM patients were employed. Incorporation of lactate was quantified using C^14^-labeled lactate. The role of MCT1, a member of the monocarboxylate transporters (MCTs), expressed on MM cells, was examined in the presence of its inhibitor (α-cyano-4-hydroxycinnamic acid: CHC) and by using gene-silencing technique.

**Results:**

MM cell lines as well as stromal cells were found to produce lactate. Incorporation of C^14^-labeled lactate into MM cells occurred in all 6 MM cell lines analyzed. Inhibition of MCT1 by using CHC or MCT1-targeting siRNA reduced lactate incorporation and caused apoptosis in MM cells. This apoptosis was enhanced when the activity of pyruvate dehydrogenase kinase was blocked by dichroloacetate. Survival of normal peripheral blood mononuclear cells was not influenced by MCT1 inhibition.

**Conclusions:**

The present data suggest that lactate is produced by MM cell lines and stromal cells, and contributes to the survival of such MM cells in autocrine or paracrine manners. Suppression of lactate incorporation by targeting MCT1 may provide a novel therapeutic strategy for MM which may be applicable for other B-cell neoplasms.

## Background

It has been reported that the growth of certain cancer cells is dependent on aerobic glycolysis to obtain ATP efficiently via glycolysis under hypoxic conditions, ultimately leading to the production of lactate by cancer cells [1]. It has also been reported that elevated lactate levels within tumors are correlated with the incidence of metastases [2,3], tumor recurrence, radioresistance [4], and poor prognosis [5].

Lactate is transported through monocarboxylate transporters (MCTs), which are composed of 14 members and encoded by the SLC16 gene family. Among the MCTs, only four isoforms (MCT1–MCT4) are known as proton-linked transporters. MCT1 has a superior affinity for lactate compared with MCT4, and therefore MCT1 is considered to facilitate lactate uptake, whereas MCT4 is considered to export lactate from inside cells [5-8]. The functional expressions of MCT1 and MCT4 are regulated by CD147 (basigin), which is essential for trafficking and anchoring MCTs in plasma membranes [9-11]. MCT1 is expressed in most tissues and various cancer cells (e.g., colon, breast, lung, prostate, stomach) [5,12], and MCT1 inhibition decreases the intracellular pH, resulting in cell death [13].

We previously reported that genes related to aerobic glycolysis are upregulated in MM cells, and that myeloma cells produce large amounts of lactate in correlation with expression of the *LDH* gene [14]. However, accumulating evidence indicates that lactate produced by cancer cells is incorporated into the cancer cells themselves as a fuel for oxidative phosphorylation [15,16]. Doherty [16] et al. reported that lactate is produced by stromal cells and supplied to oxidative cancer cells, which is known as the reverse Warburg effect. Similar finding was reported in diffuse large B-cell lymphoma by Martinez et al., showing that production of lactate from lymphoma associated stroma cells and lactate incorporation to lymphoma cells [17]. These previous reports strongly indicate that lactate is not an energy waste, but instead is actively incorporated into cells and contributes to the survival of solid tumors or B-cell neoplasms. However, to date, there have been no reports showing the incorporation of lactate into myeloma cells. Thus, in the present study, we investigated the kinetics of lactate in myeloma cells and its importance for the survival of myeloma cells.

## Results

### Expression of lactate transporters and lactate incorporation into MM cells

**Figure 1:**
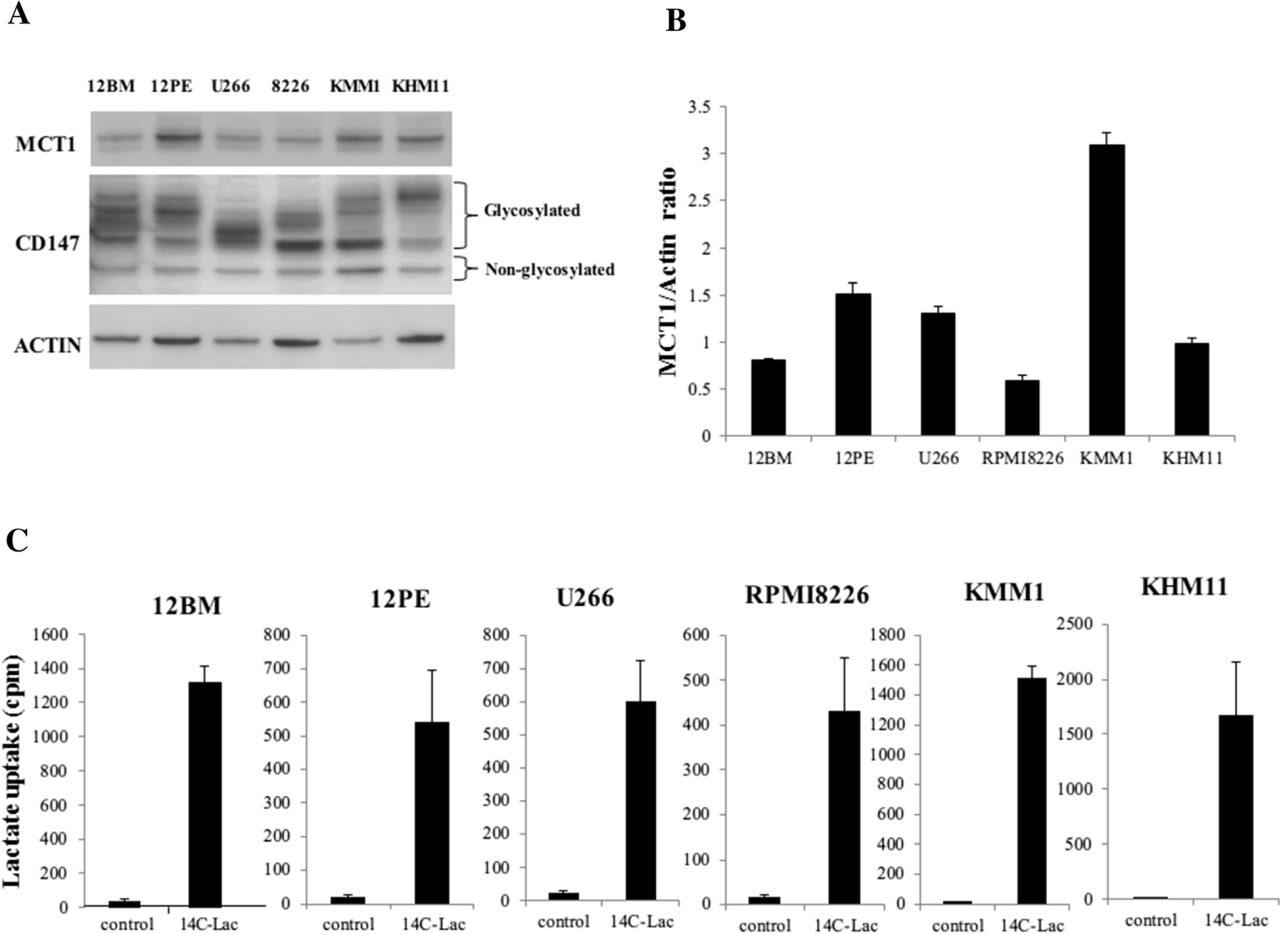
Analysis of lactate transporters and lactate kinetics in myeloma cell lines. **(A)** Western blot analyses of MCT1 and CD147. MCT1 and CD147 are found in all cell lines at various levels. **(B)** Densitometry analysis of MCT1. The staining intensities of MCT1 in **(A)** were quantified by comparison with actin. **(C)** Incorporation of lactate into myeloma cell lines. The amounts of ^14^C-lactate in myeloma cells were quantified. Lactate is clearly incorporated into all myeloma cell lines.

Expressions of MCT1 and CD147 were detected at various levels in myeloma cell lines by western blotting (Figure 1A and B). Analyses of lactate incorporation into myeloma cells showed that lactate was indeed incorporated into all myeloma cell lines at various levels (Figure 1C).

**Figure 2:**
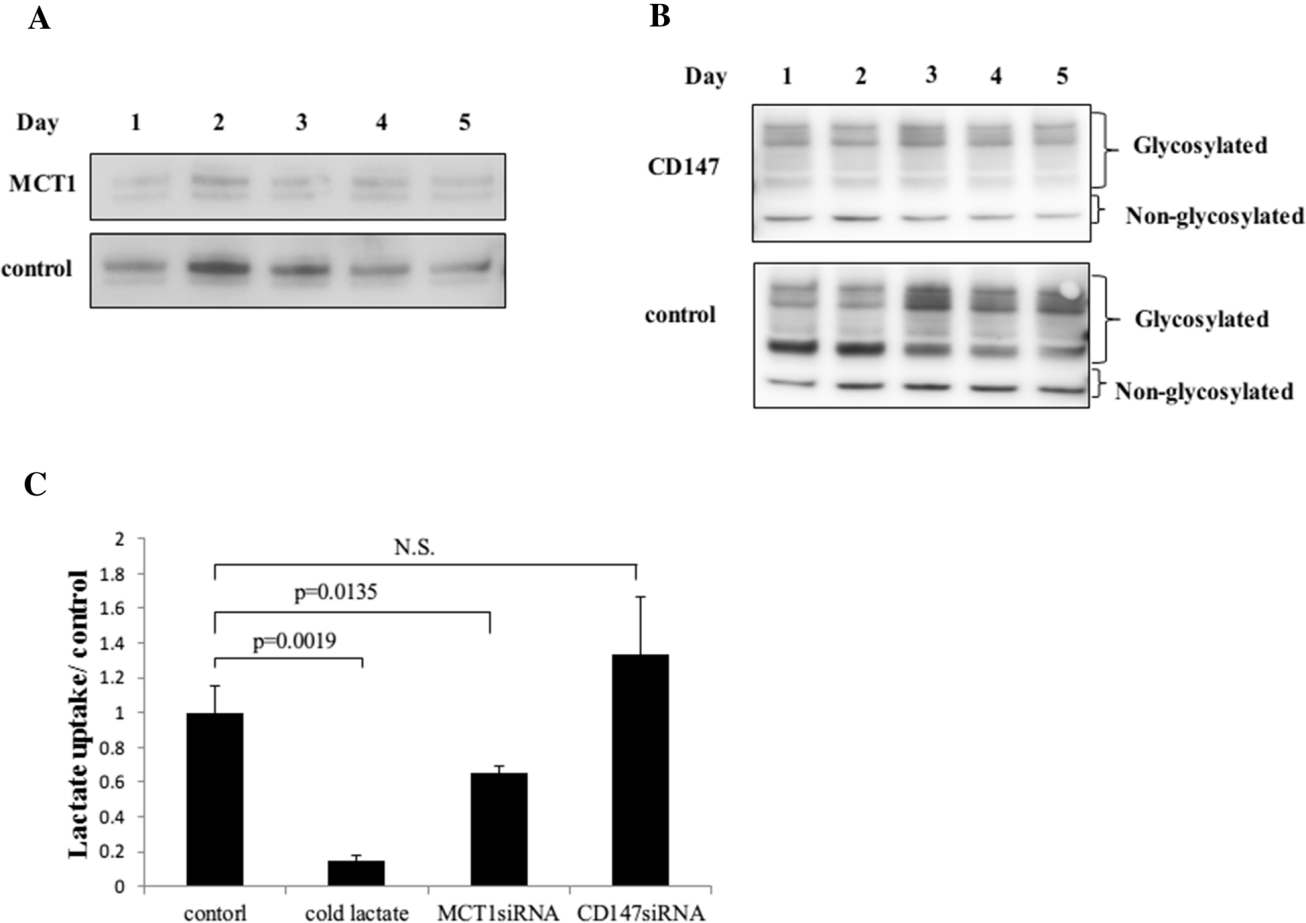
Knockdown of MCT1 induces apoptosis and reduces lactate incorporation. **(A, B)** MCT1 **(A)** and CD147 **(B)** mRNA expressions in KMM-1 cells were inhibited by siRNA transfection. The cells were treated with each siRNA and incubated for up to 5 days. Cell lysates harvested on each day were subjected to western blot analysis. Upper panel: siRNA-treated cells. Lower panel: control (untreated) cells. The siRNA-treated cells show significant reductions in MCT1 or CD147. **(C)** Lactate incorporation is inhibited by MCT1 inhibition. The siRNA-treated cells (KMM-1) were analyzed for lactate incorporation using ^14^C-lactate. To ensure the specificity of the analysis, cells were treated with radiolabeled lactate alone or combined with unlabeled lactate (cold lactate) simultaneously. Note that lactate incorporation is significantly inhibited by the siRNA for MCT1, but not by the siRNA for CD147.

Next, we analyzed how lactate was incorporated into myeloma cells. To elucidate the contribution of MCT1 to lactate incorporation, the expressions of MCT1 or CD147 were inhibited by siRNA transfection (Figure 2A and B). MCT1 knockdown led to a significant reduction in lactate incorporation, while CD147 knockdown did not result in a reduction in lactate incorporation (Figure 2C), suggesting a contribution of MCT1 to lactate incorporation into myeloma cells.

### MCT1 inhibition induces apoptosis

**Figure 3:**
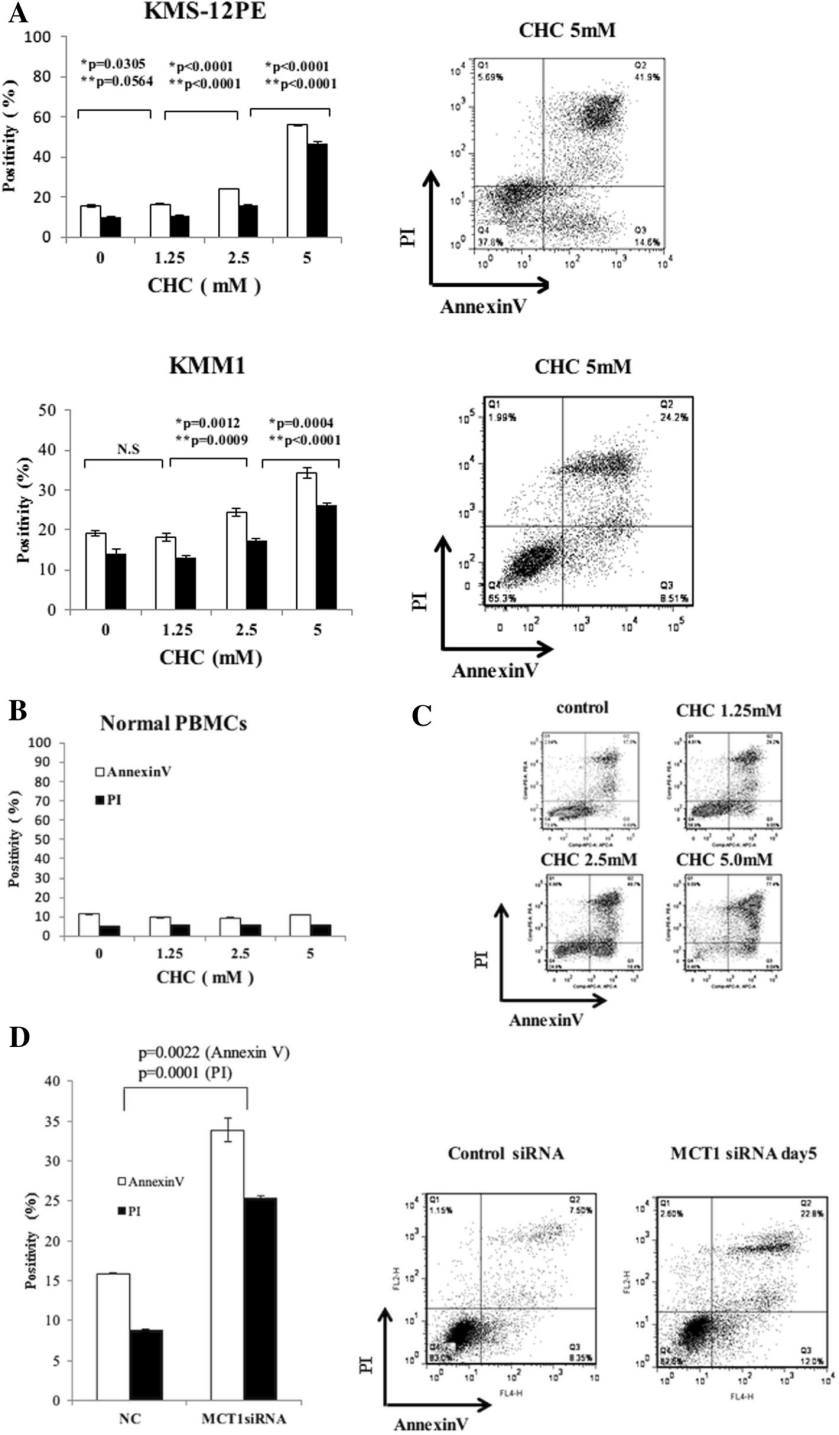
Induction of apoptosis in myeloma cells by modulation of MCT1. **(A)** Induction of apoptosis by CHC. The myeloma cell lines KMS-12PE (upper panels) and KMM-1 (lower panels) were treated with CHC at 0, 1.25, 2.5, or 5 mM for 24 h and then stained with Annexin V/PI for flow cytometry analysis. Treatment with CHC induces Annexin V-positive (open bars) and PI-positive (filled bars) populations in a dose-dependent manner. *p values for annexin V; **p values for PI. Representative raw data of the flow cytometry analyses are shown in the right panels. **(B)** CHC does not induce apoptosis in normal PBMCs. PBMCs were treated with CHC as described for (A), and do not undergo apoptosis. **(C)** CD138 positive cells obtained from a MM patient were treated with CHC at 0, 1.25, 2.5, or 5 mM for 24 h and then stained with Annexin V/PI for flow cytometry analysis. CHC induced apoptosis in myeloma cells obtained from a MM patient in a dose dependent manner. **(D)** MCT1 knockdown by siRNA results in induction of apoptosis. KMM-1 cells were treated with each siRNA and incubated for up to 5 days. On day 5, cells were subjected to Annexin V/PI staining. Amount of apoptotic cells are quantified and shown in the left panel. Results of FACS analysis are shown in the right panel. Knockdown of MCT1 shows significant induction of apoptosis.

Because we found that lactate was incorporated into myeloma cells, we hypothesized that incorporated lactate may serve as an energy resource for myeloma cells, and therefore that myeloma cells may undergo apoptosis when there is a shortage of lactate within the cells. To prove this hypothesis, we used CHC, a competitive inhibitor of MCT1, to investigate whether it induces apoptosis in myeloma cell lines by inhibiting lactate incorporation. As expected, CHC induced apoptosis in a dose-dependent manner (Figure 3A). However, CHC did not show cytotoxicity toward normal peripheral blood mononuclear cells (PBMCs) (Figure 3B). We confirmed the cytotoxic activity of CHC toward myeloma cells isolated from MM patients and found significant induction of apoptosis by CHC (Figure 3C). Subsequently, we utilized MCT1-knockdown cells to further confirm the role of MCT1 in the survival of myeloma cells. As shown in Figure 3D, significant induction of apoptosis was found upon MCT1 mRNA inhibition. However, CD147 knockout did not contribute to apoptosis (data not shown).

### Increased cytotoxic effect of CHC with a glycolysis inhibitor

**Figure 4:**
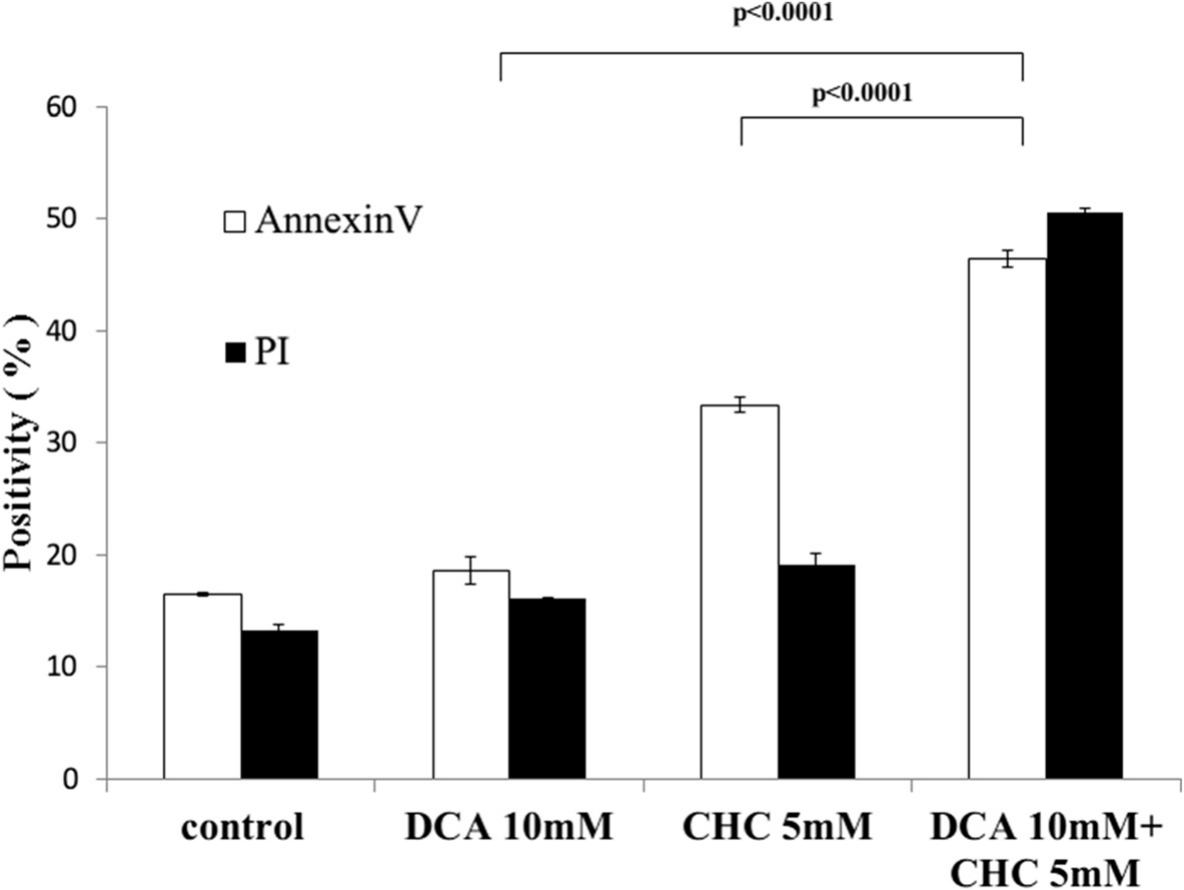
Combined treatment of CHC with DCA increases cell death. KMM1 cells were treated with CHC or DCA alone or the combination of CHC and DCA, followed by Annexin V/PI analysis. CHC and DCA each induce apoptosis alone, and the apoptosis is enhanced by the combined treatment (p < 0.0001, Annexin V or PI).

Next, we investigated the effect of combined treatment of DCA, which theoretically reduces lactate production by inhibiting pyruvate dehydrogenase kinase, with CHC. We considered that the combination of DCA with CHC should accelerate lactate reduction within myeloma cells and eventually lead to enhancement of apoptosis. As expected, this combined treatment caused a significant increase in apoptosis in the myeloma cell line KMM-1 (Figure 4).

## Discussion

**Figure 5:**
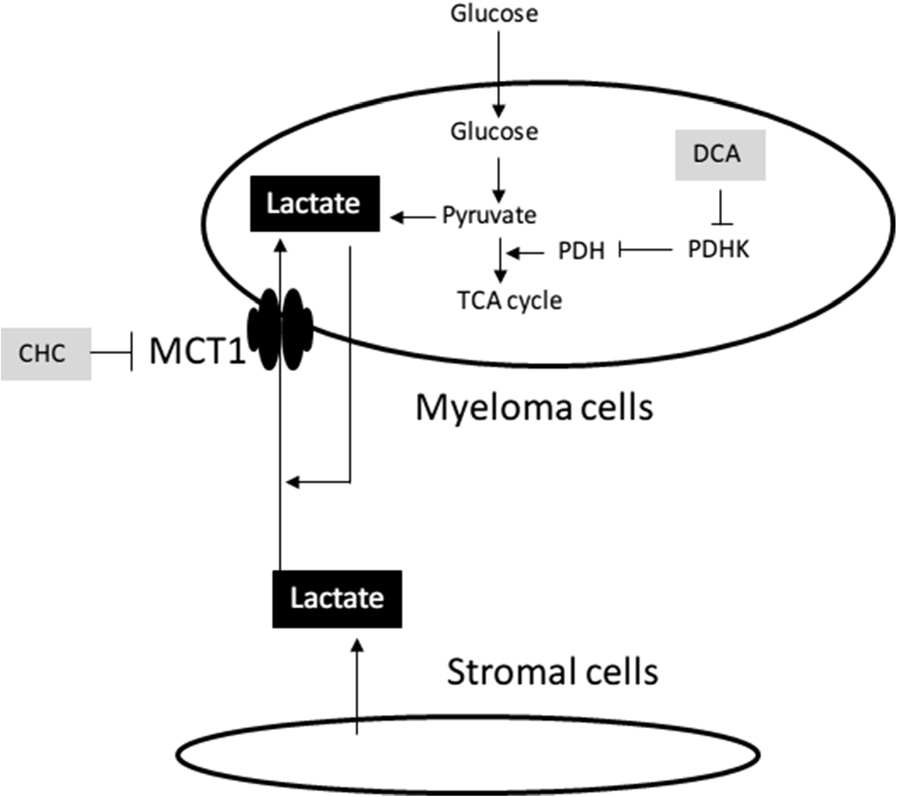
Schematic model of lactate kinetics between myeloma cells and bone marrow stromal cells. Lactate is produced by both myeloma cells and bone marrow stromal cells, suggesting that it acts as an autocrine and paracrine energy source. MCT1 may play an important role as a gate for incoming lactate. Targeting of MCT1 may result in decreased incorporation of lactate, resulting in cell death. PDH; pyruvate dehydrogenase, PDHK; pyruvate dehydrogenase kinase, MCT1; monocarboxylate transporter1, DCA; Dichloroacetate CHC; α-cyano-4-hydroxycinnamic acid.

We previously reported that aerobic glycolysis is operational in myeloma cells [14] and observed that significant amounts of lactate were produced by myeloma cells as a result of glycolysis. However, lactate is not a wasted metabolite, but can be considered as an important energy source for cancer cells [6,15,16,18,19]. Doherty et al. [16] reported that lactate is supplied to cancer cells from the surrounding environment, and referred to this phenomenon as the reverse Warburg effect. We have shown that lactate was clearly incorporated into myeloma cells and that this incorporation was mediated by MCT1. We also observed production of lactate by bone marrow stromal cells (data not shown), suggesting that the reverse Warburg effect might be applied to the microenvironment in MM as previously reported in B-cell lymphoma [17]. Taking these hypotheses together, we further suggest that lactate may serve as an autocrine energy resource, because it is both produced and incorporated by myeloma cells (Figure 5).

However, the mechanisms that regulate lactate kinetics are still not well established. We found that CD147 knockdown did not influence lactate uptake or induce apoptosis, suggesting that CD147 is not required for lactate kinetics. Additionally, we found that MCT1 knockdown decreased lactate uptake, and thus consider that lactate is incorporated by MCT1, but not through CD147. However, other investigators reported that both CD147 and MCT1 are responsible for proliferation of myeloma cells and exportation of lactate [13], suggesting that the exact roles of these molecules in lactate transport remain controversial.

Given the accumulation of reports showing MCT1 expression in various cancer cells (e.g. breast, colon, and lung cancer) and its correlation with poor prognosis [2,3,5,20,21], these previous reports indicate that MCT1 should play an important role for the survival of myeloma cells. Walters et al. [13] also reported that MCT1 mRNA expression levels were significantly higher in MM plasma cells than MGUS plasma cells, and that MCT1 expression was correlated with resistance to chemotherapy. Because MCT1 was reported to be regulated by c-Myc in some cancer cells [22-25], and MYC dysregulation was caused by complex translocation or insertion as a late progression event in MM, MCT1 could be induced in advanced MM under the control of c-Myc. Given that MCT1 could be an adverse prognostic factor, we consider that MCT1 could be a good therapeutic target for advanced MM.

CHC, a competitive inhibitor for MCT1, was reported to show anticancer effects in various cancer cells [15,26-28]. In a previous report, CHC was found to inhibit mitochondrial respiration and decrease cell growth through inhibition of cellular pyruvate uptake [29]. Zhao et al. [26] reported that CHC suppressed tumor growth of osteosarcoma in vitro and in vivo through regulation of the NF-kB pathway. Moreover, they reported that CHC enhanced the efficacy of conventional chemotherapeutic agents. Another report showed that CHC contributed to chemosensitization against cisplatin in a colon cancer cell line [28]. However, there are no reports showing antitumor effects of CHC for myeloma cells. In this study, CHC induced apoptosis in myeloma cells, but did not exhibit cytotoxicity toward normal PBMCs, indicating that MCT1 could be a safe therapeutic candidate molecule. Moreover, because we found augmentation of cytotoxicity by CHC with DCA, cooperative inhibition of the glucose metabolic pathway could be a useful option.

Our results indicate that modification of lactate kinetics, such as targeting of MCTs by antibody-based chemotherapeutic reagents, could be an attractive modality to control the growth of myeloma cells although the target of this modality may be limited for some subsets of MM cases in which MM cells are depending on Reverse Warburg Effect. Because lactate does not seem to provide to a large contribution for resting normal cells, this modality should provide efficacy that is relatively specific to cancer cells with minimal toxic effects toward normal tissues.

## Conclusions

We present here that lactate is produced by MM cell lines and stromal cells, and contributes to the survival of such MM cells in autocrine or paracrine manners. Lactate incorporation is dependent on MCT1. Suppression of lactate incorporation by targeting MCT1 may provide a novel therapeutic strategy for MM.

## Materials and methods

### Cells and cell culture

Human myeloma cell lines KMS-12BM (12BM) [30], KMS-12PE (12PE) [30], U266 [31], RPMI8226 [32], KMM-1 [33], and KHM11 [34] were cultured in RPMI-1640 containing 10% FBS at 37°C under 5% CO2. Usage of isolated myeloma cells from bone marrow samples were approved by Ethical Committee of Kumamoto University.

### Inhibitors

Dichloroacetate (DCA) and α-cyano-4-hydroxycinnamic acid (CHC) were purchased from Sigma-Aldrich (St Louis, MO, USA), and dissolved in phosphate-buffered saline and dimethyl sulfoxide (DMSO), respectively.

### Measurement of lactate

Lactate concentrations were evaluated using a lactate meter (Lactate Pro2; Arkray, Kyoto, Japan), which electronically analyzed potassium ferrocyanide converted from ferricyanide by lactate.

### cDNA synthesis and real-time PCR

RNA was extracted from myeloma cells using TRIzol reagent (Invitrogen, Carlsbad, CA, USA). cDNA synthesis was performed using a SuperScript First-Standard Synthesis System (Invitrogen) according to the manufacturer’s protocol. Quantitative PCR analyses were performed with Assay-on- Demand primers and Taqman Universal PCR Master Mix Reagent (Applied Biosystems, Foster City, NJ, USA). The samples were analyzed using an ECO™ Real-Time PCR System (Illumina, San Diego, CA, USA). The ΔΔCt method was employed to analyze the relative changes in gene expression as previously described [35], with *ACTB* as a normalization control. The following primers were used to quantify the expression of MCT1 and actin, respectively: *SLC16A1* (Hs00161826_m1); and *ACTB* (Hs99999903_m1).

### Western blot analysis

Cell lysates were prepared using M-PER Mammalian Protein Extraction Reagent (Pierce Biotechnology Inc., Rockford, IL, USA) after addition of Halt EDTA-free phosphatase inhibitor cocktail and Halt protease inhibitor cocktail (both from Pierce Biotechnology Inc.). Quantification of total protein was performed with a Pierce BCA Protein Assay Kit (Thermo Scientific, Waltham, MA, USA), and equal amounts of protein were used for analysis. The cell lysates were separated in NuPAGE Bis-Tris precast gels (Invitrogen) and transferred to PVDF membranes. The membranes were blocked with 5% non-fat dry milk dissolved in Tris-buffered saline (TBS) containing 0.5% Tween-20 (TBS-T) for 1 h at room temperature, and then incubated with primary antibodies at 4°C for 18 h. The following primary antibodies were used: anti-MCT1 (Santa Cruz Biotechnology, Santa Cruz, CA, USA; sc-365501; 1:500), anti-CD147 (Cell Signaling Technology, Beverly, MA, USA; #12314; 1:250), and anti-ACTB (Santa Cruz Biotechnology; sc-8432; 1:1000). After washing with TBS-T, the membranes were incubated with a horseradish peroxidase-conjugated secondary antibody (GE Healthcare, Little Chalfont, UK) for 1 h at room temperature. The antibody-bound proteins were visualized using ECL-prime Western Blotting Detection Reagent (GE Healthcare) and an LAS-1000 Bio-image Analyzer (GE Healthcare).

The intensity of the western blot signals was quantified by densitometry using image j.

### Analysis of apoptosis

The myeloma cell lines were incubated in the presence of DCA or CHC for 24 h. Apoptosis in the myeloma cell lines was quantified by staining with Annexin V-allophycocyanin (Molecular Probes, Eugene, OR, USA) and propidium iodide (PI) (MBL, Nagoya, Japan). The samples were analyzed by flow cytometry (FACSCalibur or FACSVerse; Becton Dickson, San Jose, CA, USA).

### RNA interference experiments

An oligonucleotide targeting human SLC16A1 (5′-CAGCAGTATCCTGGTGAATAA-3′; siSLC16A1) was purchased from Qiagen (Valencia, CA, USA). Transfections were performed using Hiperfect Transfection Reagent (Qiagen) according to the manufacturer’s protocol. Briefly, 2 × 10^5^ KMM-1 cells in 100 μL of medium were seeded into 24-well plates and transfected with 750 ng of siSLC16A1 or control siRNA.

### Lactate uptake assay

On day 2 after siRNA transfection, KMM-1 cells were incubated in uptake buffer (10 mM HEPES, 5 mM KCl, 150 mM NaCl, 1 mM MgCl_2_, pH 7.5) containing ^14^C-lactate (PerkinElmer, Waltham, MA, USA) for 1 h at 37°C. Uptake was stopped by incubating the cells at 4°C, and the cells were washed twice using 200 μL of uptake buffer. The cells were then lysed in 200 μL of uptake buffer containing 1% SDS and the cell lysates were collected into scintillation vials for quantification of ^14^C-lactate uptake. To ensure selective incorporation of lactate, some cells were simultaneously treated with ^14^C-lactate and unlabeled lactate (10 mM).

### Statistical analysis

The data were analyzed statistically by Student’s *t*-test using Statflex version 6 (Artech Co. Ltd., Osaka, Japan). Values of p < 0.05 were considered statistically significant.

## Abbreviations

(MM): Multiple myeloma
(MCTs): Monocarboxylate transporters
(CHC): α-cyano-4-hydroxycinnamic acid
(DCA): Dichloroacetate
(DMSO): Dimethyl sulfoxide
(TBS): Tris-buffered saline
